# Unraveling the Hidden Heterogeneities of Breast Cancer Based on Functional miRNA Cluster

**DOI:** 10.1371/journal.pone.0087601

**Published:** 2014-01-30

**Authors:** Li Li, Chang Liu, Fang Wang, Wei Miao, Jie Zhang, Zhiqian Kang, Yihan Chen, Luying Peng

**Affiliations:** 1 Division of Medical Genetics, Tongji University School of Medicine, Shanghai, China; 2 Key Laboratory of Arrhythmias, Ministry of Education, Tongji University, Shanghai, China; Memorial Sloan Kettering Cancer Center, United States of America

## Abstract

It has become increasingly clear that the current taxonomy of clinical phenotypes is mixed with molecular heterogeneity, which potentially affects the treatment effect for involved patients. Defining the hidden molecular-distinct diseases using modern large-scale genomic approaches is therefore useful for refining clinical practice and improving intervention strategies. Given that microRNA expression profiling has provided a powerful way to dissect hidden genetic heterogeneity for complex diseases, the aim of the study was to develop a bioinformatics approach that identifies microRNA features leading to the hidden subtyping of complex clinical phenotypes. The basic strategy of the proposed method was to identify optimal miRNA clusters by iteratively partitioning the sample and feature space using the two-ways super-paramagnetic clustering technique. We evaluated the obtained optimal miRNA cluster by determining the consistency of co-expression and the chromosome location among the within-cluster microRNAs, and concluded that the optimal miRNA cluster could lead to a natural partition of disease samples. We applied the proposed method to a publicly available microarray dataset of breast cancer patients that have notoriously heterogeneous phenotypes. We obtained a feature subset of 13 microRNAs that could classify the 71 breast cancer patients into five subtypes with significantly different five-year overall survival rates (45%, 82.4%, 70.6%, 100% and 60% respectively; *p* = 0.008). By building a multivariate Cox proportional-hazards prediction model for the feature subset, we identified has-miR-146b as one of the most significant predictor (*p* = 0.045; hazard ratios = 0.39). The proposed algorithm is a promising computational strategy for dissecting hidden genetic heterogeneity for complex diseases, and will be of value for improving cancer diagnosis and treatment.

## Background

The patients with similar prognosis often respond differently to the same treatment. One explanation for this is that disease with similar phenotypes may have different genetic causes, a phenomena of genetic heterogeneity. Such genetic heterogeneity presents a significant challenge for modern clinical practice and biomedical research on common human diseases. MicroRNAs are a class of small noncoding RNAs that regulate translation of protein coding mRNAs by translational inhibition or cleavage of the mRNA transcripts. In humans, miRNAs are approximately 22-nucleotide-long single-stranded RNAs[1]. They play a key role in the post-transcriptional regulation of up to 30% of protein-coding genes, and have a profound impact on many cellular processes during development and adult life.

MicroRNA(MiRNA) expression profiles of tumour samples have recently been shown to provide phenotypic signatures for specific types of cancer [2]–[6], making it potentially useful in tackling the heterogeneity issues for complex human diseases. Recent studies have used DNA microarrays to study breast cancer, and have shown that it was possible to identify subgroups of patients in terms of different survival courses by gene expression profiles [7]. Blenkiron *et al*. [8] analysed the expression of miRNAs in breast tumour samples using a bead-based flow-cytometric profiling method. To our knowledge, this was the first integrated analysis of miRNA expression, mRNA expression and genomic changes in breast cancer. They showed that miRNAs could act as molecular signature to distinguish the subtypes of breast cancer, which would be unlikely to be discovered by traditional clinical approaches. They further identified distinctive microRNA signatures that correlated with cytogenetic and molecular subtypes of breast cancer. However, most methods that aimed to identify clinically relevant subtypes using microRNAs usually employed unsupervised learning techniques, such as hierarchical clustering, which would be of limited use when the disease heterogeneity results from only a small subset of the miRNAs participating in a particular cellular process. In these cases, the “signal” or relevant miRNAs may be overwhelmed by the “noise” generated by the vast majority of unrelated data.

In this study, we aimed to identify a subset of miRNAs that could dissect breast cancer patients with different survival outcomes. We employed a coupled two-way clustering algorithm (CTWC) [9]–[11] to iteratively partition breast cancer samples and microRNA sets. The partition was done by a super-paramagnetic clustering (SPC) algorithm [12] that can automatically determine the number of partitions, and generate robust stable phenotypic partitions and the significant feature signatures (miRNA subsets). For identifying the optimal miRNA cluster, we developed a functional consistency index to evaluate the functional consistency of the significant miRNA subsets. The index considers not only expression correlation but also chromosome distance between miRNAs within a significant miRNA subset. The index was developed based on the following considerations. With this measurement, the optimal miRNA cluster is expected to not only dissect breast cancer samples efficiently, but also function in a coherent manner. Finally we applied the proposed method to a publicly available breast cancer microRNA expression profiling dataset, and then employed the Kaplan-Meier survival analysis [13] and multivariate Cox proportional-hazards prediction modelling [14] to determine the differential survival outcomes of new subtypes.

## Materials and Methods

### Dataset Preprocessing

A miRNA expression data (GSE7842) was download from the Gene Expression Omnibus (GEO)(http://www.ncbi.nlm.nih.gov/geo/). The raw dataset consisted of the expression profiles of 309 miRNAs in 137 samples in which 119 passed quality control (93 primary human breast tumours, 21 cell lines and five normal breast samples). The original breast cancer study used a single sample predictor (SSP) to assign individual samples to one of five breast tumour subtypes: Luminal A(ER+, PR+, HER2−), Luminal B(ER+, PR+, HER2+), Basal-like(ER−, PR−, HER2−), HER2+(ER−, PR−, HER2+) and Normal breast-like [8]. For heterogeneity analysis, we used the subset of the data that consists of the expression profiles of 133 miRNAs, which express in 71 human breast cancers with specific clinical information (Menopause stage, Size, Grade, Total nodes, NPI (Nottingham Prognostic Index), Survival time, Dead, DFI and Age etc.) for subsequent analysis.

### Coupled two-way clustering

The applied CTWC algorithm is a heuristic and iterative method, and is implemented by a stand-alone package. Super-Paramagnetic Clustering was used as the underlying clustering tool to partition the whole dataset into subsets of miRNA and samples iteratively until significant partitions (submatrices) are obtained. A detailed description of the SPC and CTWC algorithm can be found in [15]. For a microRNA expression profile matrix 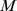, we denoted the initial sample set as 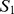, and the microRNA set 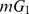. Clustering microRNA set 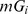 on the basis of their expression levels over the set of samples 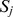 was referred to the process in an operation denoted by 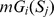. Similarly defined, 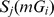 described the process of clustering 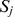 using all microRNA of 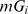. We employed CTWC for identifying significant miRNA subsets in the breast cancer dataset. Specifically, first, we clustered all samples using all miRNAs to identify stable sample partitions, and clustered all miRNAs using all samples to identify stable miRNA subsets. Then, we clustered the miRNA gained in the previous step using the newly defined sample partitions (including all samples) to find the responsible miRNA subsets of high discriminating power. Finally, we clustered each sample partition again using each miRNA subset with high discriminating power. In the searching process, we explored the cluster depth for both dimensions of samples and miRNAs. The cluster depth selected was based on the empirical judgement on whether the clinical samples could be well separated using the candidate miRNA subset(s).

### Evaluation of a miRNA subset using the functional consistency score

We obtained many high-correlation sample partitions and miRNA subsets by CTWC. To evaluate the power of dissecting tumour subtypes, we developed a functional consistency score that evaluates the biological significance of a miRNA subset. The steps for computing a functional consistency score of miRNA subsets 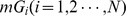
were as follows:

**(1) Correlation coefficient (CC).** For each 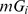, we computed the correlation coefficient
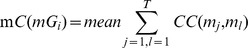
where 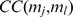
is the Pearson correlation coefficient between microRNA 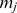 and 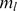 in 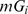, and 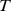 is the number of microRNA in 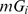
_._

**(2) Computing the chromosome cluster.** Based on the chromosome location of each miRNA in human via MiRGen [16], we grouped human miRNAs into different clusters by requiring that in each cluster the maximum distance between any two miRNAs be less than 50K bp, and obtained 51 intergenic or gene-resident spatial clusters in which 38 overlap with the 133 miRNAs in this study.

For each pair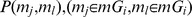, we map the pair 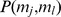 to 51 chromosome clusters. Then, we computed the number of the pair 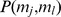 that overlaps the 51 chromosome clusters 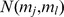. The chromosome consistency score of 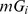 is then defined as:
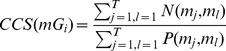
where 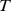 is the total number of microRNA in 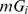.

**(3) Functional consistency.** For each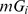,
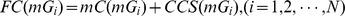



A higher 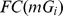 corresponds to a higher degree of functional consistency among the miRNAs involved in 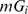.

### Survival analysis

To verify the clinical significance of the identified hidden breast cancer subtypes, we plotted their survival curves by Kaplan–Meier product-limit method, and assessed the differences between the survival curves of breast cancer patients belonging to different subtypes by log-rank test. The multivariate Cox proportion-hazards model was used to predict the overall survival time, and to determine the significance (at significant level *p*<0.05) of the effects if the miRNAs is included in the identified miRNA subset(s) on the patient' survival months. Wald Chi-square test was used to determine the significance of each predictor's hazard toward the survival time.

### Predicting the targets of optimized miRNA subset

To further understand the function and dissect the involved pathways in the optimized miRNAs, we predicted the targets of the optimized miRNA subset 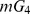 using mirGen [16] which includes miRanda, PicTar and TargetScanS prediction programs. This is based on Sethupathy *et al*' study [17], which found that the intersection of the predictions from prediction program could achieve both high sensitivity and specificity. In addition, we also considered the expression of predicted target genes of miRNAs. Those genes with anti-correlated expression of the miRNAs were filted and mapped to KEGG in order to study the underlining related pathways.

### Computational algorithms

**Figure 1:**
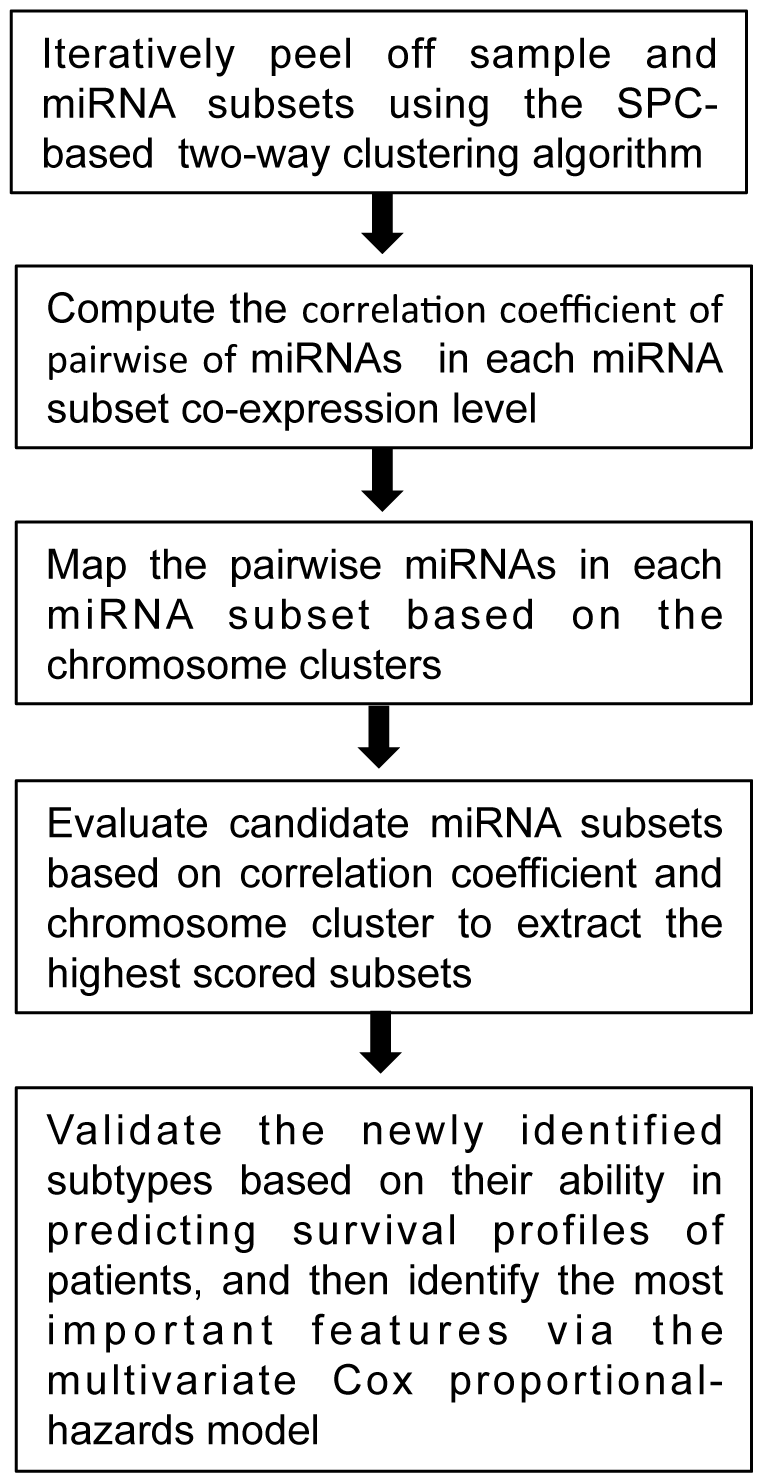
The graphic algorithm flow for the proposed SPC-based two-way clustering.

The flowchart of the proposed method was graphically depicted by Figure 1. The programming codes for computing a function consistency score are available upon request to the authors. The hierarchical dendrogram resulted from the coupled two-way clustering was plotted by Treeview[18], [19].

## Results

### Selection of miRNA subset that partitions breast cancer samples with highest functional consistency score

We employed CTWC algorithm to search for significant miRNA subsets that could distinguish breast cancer subtypes. CTWC identified many highly correlated miRNA subsets during the recursive partitioning of samples and miRNAs. In this study we aimed to identify the partition of breast cancers [8]. With CTWC, we identified 75 stable and significant miRNA subsets at the cluster depth of one 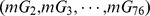 (Table 1). Then, we determined the function consistency scores of the 75 stable miRNA subsets separately.

**Table 1:** miRNA clusters using CTWC

Objects	Clusters																		
mG1(s1)	mG_2_	mG_3_	mG_4_	mG_5_	mG_6_	mG_7_	mG_8_	mG_9_	mG_10_	mG_11_									
mG1(S2)	mG_12_	mG_13_	mG_14_	mG_15_	mG_16_	mG_17_	mG_18_	mG_19_	mG_20_	mG_21_	mG_22_	mG_23_	mG_24_	mG_25_	mG_26_	mG_27_	mG_28_	mG_29_	
mG1(S3)	mG_30_	mG_31_	mG_32_	mG_33_	mG_34_	mG_35_	mG_36_	mG_37_	mG_38_	mG_39_	mG_40_								
mG1(S4)	mG_41_	mG_42_	mG_43_	mG_44_	mG_45_	mG_46_	mG_47_	mG_48_											
mG1(S5)	mG_49_	mG_50_	mG_51_	mG_52_	mG_53_	mG_54_	mG_55_												
mG1(S6)	mG_56_	mG_57_	mG_58_	mG_59_	mG_60_	mG_61_													
mG1(S7)	mG_62_	mG_63_	mG_64_	mG_65_	mG_66_	mG_67_	mG_68_	mG_69_	mG_70_	mG_71_	mG_72_	mG_73_	mG_74_	mG_75_	mG_76_				

We computed and sorted the correlation coefficient of 75 miRNA subsets. The CC (the average Pearson correlation coefficient across all pairs of miRNAs in the cluster, see Methods for details) of the top 5 subsets 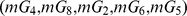
are: 0.565230769, 0.55772, 0.50603, 0.48114, 0.47562, respectively. 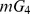
has the highest CC. The CC of pairwise miRNAs in 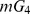
is shown in Table S1.

For 5 microRNA subsets with higher CC, we mapped all combinations of microRNAs pairs in each microRNA subset into 51 different chromosome clusters based on MiRGen, and then computed a chromosome consistency score for each miRNA subset. We found that score of 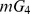
was 2/78 = 0.0256. The other 4 microRNA subsets have no miRNAs pairs belonging to any chromosome cluster. Based on the correlation coefficient and chromosome cluster of miRNA subsets, we found the has 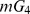
the highest functional consistency score with 0.5908.

Among the 13 miRNAs in 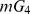, miR-221/222 negatively regulates estrogen receptor alpha, and is associated with tamoxifen resistance in breast cancer [20]. The expression of miR-206 is down-regulated in estrogen receptor alpha-positive human breast cancer [21]. Recent data [22] indicated that the pattern of expression of miR-146a and miR-146b was similar, suggesting that their target genes might be coregulated, although they may be located on different chromosomes. Furthermore, the expression of miR-146a/b is high in those samples that have been classified as Basal-like. In the Basal-like cell lines with the highest miR-146a/b expression level, the amount of BRCA1 was particularly low. Further analysis revealed that the expression levels of miR-146b was significantly elevated only compared to Luminal B and Basal-like subtype. In addition, miR-143/145 microRNAs were repressed in Basal-like compared to Luminal subtype [23]. Lu *et al* [24] demonstrated that the expression level of miR-155 was inversely correlated with estrogen receptor. Our results are consistent with the recent discoveries.

### Clustering breast cancer samples using the selected miRNA subsets

**Figure 2:**
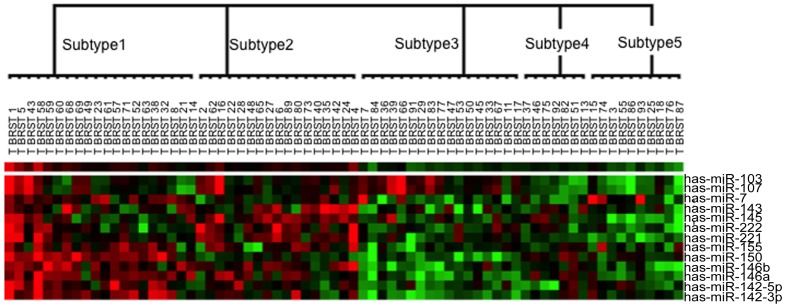
The five partitions of breast cancer were identified using 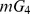 as the disease feature set in the breast cancer dataset. In the figure, each microRNA corresponds to a row, and each breast cancer sample corresponds to column. The 71 breast cancer samples were divided into five subtypes (Subtype 1, Subtype 2, Subtype 3, Subtype 4 and Subtype 5). Red areas indicate increased expression, and green areas decreased expression. Each column represents a single breast cancer sample, and each row represents a single microRNA. The dendrogram at the top shows the degree to which each breast cancer subtype is related to the others with respect to microRNA expression.

We applied 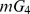 using SPC to cluster 71 breast samples from Blenkiron *et al*. using SPC [8]. Here, the Euclidean distance and Pearson's correlation coefficient were used as the sample and the miRNA expression similarity measures, respectively. We successfully partitioned the 71 samples into five subtypes (Figure 2).

**Figure 3:**
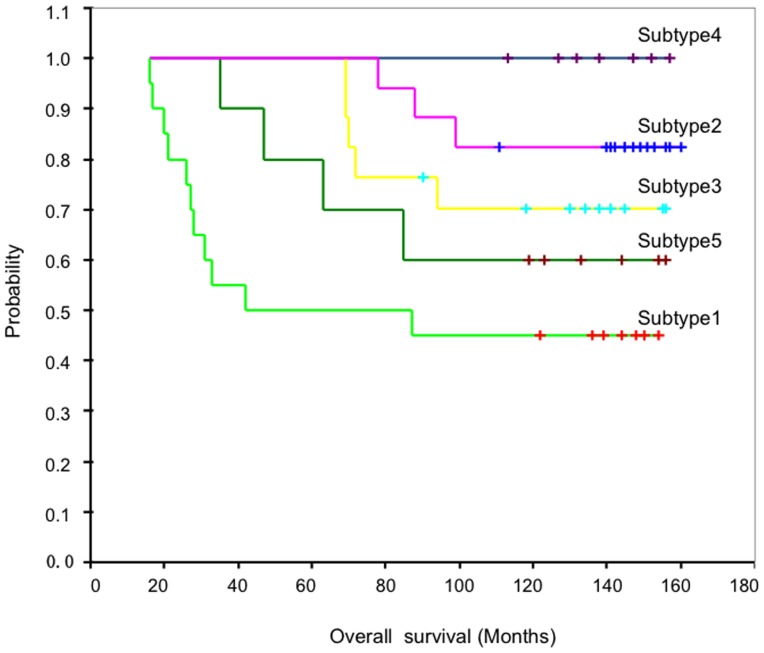
Survival curves for five subtypes of the breast cancer patients in the breast cancer dataset.

To verify the clinical significance of the identified hidden breast cancer subtypes, we plotted the survival curves by Kaplan-Meier product-limit method, and assessed the differences between the survival curves of breast cancer patients with different subtypes by a log-rank test (Figure 3). The 5 year survival rates for five subtypes were 45%, 82.4%, 70.6%, 100% and 60% (*p* = 0.008), respectively.

In order to develop a compact model for clinical use, we further identified miRNAs that contributed mostly to the high prediction power. Multivariate Cox proportional-hazards model was used to analyze miRNAs in 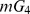. To reduce the number of variables to be modeled, we applied the stepwise variable selection option (with the same inclusion and exclusion *p* value of 0.05) for the multivariate Cox proportional-hazards regression model. We found one predictor (miRNAs) in 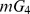 (Table 2). miR-146b was selected to be the significant prognostic predictor because of its importance involved in the underlying pathogenic mechanisms for breast cancer. We also used the Cox model to select significant predictor for ER+ and ER-. In surprise, the miR-146b was also found to be the best predictor (*p = 0.038*).

**Table 2:** Multivariate Cox proportional-hazards analysis based on the 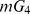
signature microRNAs relevant to survival time.

Variable	Estimated coefficient	Wald 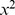	p value	Hazard ratio(95%CI)
hsa-miR-146b	-3.232	4.021	0.045	0.039(.002–.930)

### Predicting the function microRNAs in 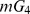


We used the Target-Function-Expression Module of miRGen to predict the targets of each microRNA in 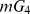
and obtained miRNA-mRNA pairs. In addition, The corresponding mRNA expression profiling of 71 samples and 19,061 mRNAs (http://www.ebi.ac.uk/arrayexpress/experiments/E-UCON-1/) was downloaded and 5228 pre-proceeded mRNAs [8] was selected after normalization. The Pearson's Correlation coefficient of each miRNA-mRNA pair was computed. Then, anti-association(CC<−0.4) pairs were regarded as reliability. Here, we only provided the results of miR-146b targets. The number of predicted miRNA-mRNA pairs by miRGen is 1,311, among which 664 have information of mRNA expression. 76 miRNA-mRNA pairs were anti-correlation in expression with CC<−0.4 in Table S2. In these mRNAs, we also mapped all reliable targets to KEGG by DAVID database [25], and found that the target genes of miR-146b mainly take part in MAPK signaling pathway, Toll-like receptor signaling pathway and Pathway in cancer. The targets of other miRNAs in 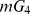, such as miR-146a, miR-221 and miR-222, were also found to be involved in Toll-like receptor signaling pathway, indicating that this pathway may be associated with the subtyping of breast cancer.

## Discussion and Conclusions

Increasing evidence have suggested that miRNAs are involved in cancer development through regulating distinct biological processes, including cellular growth and proliferation, cellular movement and migration, extra cellular matrix degradation. Though the expression level of miRNAs is generally low in cancer, their unique profiles may have significant clinical outcomes, especially for the phenotypes of cancer. Recently, many studies have shown that the abnormal expression of miRNAs is correlated with human breast cancer. In this study we showed that the two-way clustering algorithm could result in an improved prognostic accuracy over the breast cancer patients' survival profiles.

Computational discoveries of the hidden subtypes for a complex disease have to be verified by some means, e.g., a functional assay using bioinformatics approaches or a clinical validation using epidemiological approaches such as survival analysis. In unsupervised clustering analysis, however, identifying the best subset for dissecting clinically heterogeneous disease can be a challenging task as no cross-validation can be done internally. The underlying assumption for a clustering algorithm is that microRNA with similar expression patterns and adjacent chromosome location are more likely to have a similar biological function(s). However, a clustering algorithm itself does not provide proof of the best grouping of microRNA in terms of biological functions. Thus, the biological interpretation of the disease clustering results relies heavily on the expert knowledge which often may be subjective [26]. Therefore, in this study, we designed a functional consistency score for evaluating a candidate miRNA subset in terms of functional consistency. In terms of the better-characterized functionality of subset 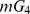 and based on the significantly different survival results for the patients defined by the newly defined subtypes, the applied two-way clustering algorithm has been demonstrated to be a feasible and promising toolbox for peeling off molecular heterogeneities of complex human diseases.

Many methods use all the microRNAs on chips or a large number of microRNAs to predict patient survival. Since the vast majority of the microRNAs in a given dataset are irrelevant to the survival of the studied patients, this may reduce the prediction accuracy of the model because of the added noise. Hence, McLachlan *et al*. [27] proposed a mixture model-based approach to the clustering of microarray expression data. In this study, we applied an integrative approach that combines a SPC-based two-way clustering with a functional consensus to identify the functionally sounding and the most compact subset of microRNAs underlying the phenotypic partitions of patients.

Application of the proposed approach to breast cancer datasets led to identification of microRNA subsets, and further multivariate Cox proportional-hazards modeling defined microRNA-146b as one significant predictor for the survival of the breast cancer patients in the dataset. The individual miRNAs have only limited impact on their targets and multiple miRNAs are needed to drastically reduce transcription levels of target. We also found the targets of serval microRNAs in the optimal miRNA subset involoved similar pathways. Overall, our results demonstrated that the proposed approach is highly promising for peeling off the hidden genetic heterogeneity based on modern omics data, and may lead to an improved diagnosis and treatment of cancers.

## Supporting Information

Table S1
**The correlation coefficient of among miRNAs in** 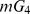 **.**
(DOCX)

Table S2 **The anti-association miRNA-mRNA of among miR-146b.**
(DOCX)

## References

[1] Bartel DP (2004) MicroRNAs: genomics, biogenesis, mechanism, and function. Cell 116: 281–297. 14744438 10.1016/s0092-8674(04)00045-5

[2] Lu J, Getz G, Miska EA, Alvarez-Saavedra E, Lamb J, et al (2005) MicroRNA expression profiles classify human cancers. Nature 435: 834–838. 15944708 10.1038/nature03702

[3] Kent OA, Mendell JT (2006) A small piece in the cancer puzzle: microRNAs as tumor suppressors and oncogenes. Oncogene 25: 6188–6196. 17028598 10.1038/sj.onc.1209913

[4] Thum T, Gross C, Fiedler J, Fischer T, Kissler S, et al (2008) MicroRNA-21 contributes to myocardial disease by stimulating MAP kinase signalling in fibroblasts. Nature 456: 980–984. 19043405 10.1038/nature07511

[5] Cummins JM, Velculescu VE (2006) Implications of micro-RNA profiling for cancer diagnosis. Oncogene 25: 6220–6227. 17028602 10.1038/sj.onc.1209914

[6] Cummins JM, He Y, Leary RJ, Pagliarini R, Diaz LA Jr, et al (2006) The colorectal microRNAome. Proceedings of the National Academy of Sciences of the United States of America 103: 3687–3692. 16505370 10.1073/pnas.0511155103 PMC1450142

[7] Perou CM, Sorlie T, Eisen MB, van de Rijn M, Jeffrey SS, et al (2000) Molecular portraits of human breast tumours. Nature 406: 747–752. 10963602 10.1038/35021093

[8] Blenkiron C, Goldstein LD, Thorne NP, Spiteri I, Chin SF, et al (2007) MicroRNA expression profiling of human breast cancer identifies new markers of tumor subtype. Genome biology 8: R214. 17922911 10.1186/gb-2007-8-10-r214 PMC2246288

[9] Getz G, Domany E (2003) Coupled two-way clustering server. Bioinformatics 19: 1153–1154. 12801877 10.1093/bioinformatics/btg143

[10] Getz G, Gal H, Kela I, Notterman DA, Domany E (2003) Coupled two-way clustering analysis of breast cancer and colon cancer gene expression data. Bioinformatics 19: 1079–1089. 12801868 10.1093/bioinformatics/btf876

[11] Getz G, Levine E, Domany E (2000) Coupled two-way clustering analysis of gene microarray data. Proceedings of the National Academy of Sciences of the United States of America 97: 12079–12084. 11035779 10.1073/pnas.210134797 PMC17297

[12] Tetko IV, Facius A, Ruepp A, Mewes HW (2005) Super paramagnetic clustering of protein sequences. BMC bioinformatics 6: 82. 15804359 10.1186/1471-2105-6-82 PMC1084344

[13] Kopycka-Kedzierawski DT, Billings RJ (2004) A longitudinal study of caries onset in initially caries-free children and baseline salivary mutans streptococci levels: a Kaplan-Meier survival analysis. Community Dent Oral Epidemiol 32: 201–209. 15151690 10.1111/j.1600-0528.2004.00153.x

[14] Cox DR (1972) Regression models and lifetables. JRStatSoc[B] 34: 187–220.

[15] Zhang W, Li L, Li X, Jiang W, Huo J, et al (2007) Unravelling the hidden heterogeneities of diffuse large B-cell lymphoma based on coupled two-way clustering. BMC genomics 8: 332. 17888167 10.1186/1471-2164-8-332 PMC2082044

[16] Alexiou P, Vergoulis T, Gleditzsch M, Prekas G, Dalamagas T, et al.. (2010) miRGen 2.0: a database of microRNA genomic information and regulation. Nucleic Acids Res 2010:38(Database issue):D137–41. 10.1093/nar/gkp888 PMC2808909 19850714

[17] Sethupathy P, Megraw M, Hatzigeorgiou AG (2006) A guide through present computational approaches for the identification of mammalian microRNA targets. Nature methods 3: 881–886. 17060911 10.1038/nmeth954

[18] Alon U, Barkai N, Notterman DA, Gish K, Ybarra S, et al (1999) Broad patterns of gene expression revealed by clustering analysis of tumor and normal colon tissues probed by oligonucleotide arrays. Proceedings of the National Academy of Sciences of the United States of America 96: 6745–6750. 10359783 10.1073/pnas.96.12.6745 PMC21986

[19] Eisen MB, Spellman PT, Brown PO, Botstein D (1998) Cluster analysis and display of genome-wide expression patterns. Proceedings of the National Academy of Sciences of the United States of America 95: 14863–14868. 9843981 10.1073/pnas.95.25.14863 PMC24541

[20] Zhao JJ, Lin J, Yang H, Kong W, He L, et al (2008) MicroRNA-221/222 negatively regulates estrogen receptor alpha and is associated with tamoxifen resistance in breast cancer. The Journal of biological chemistry 283: 31079–31086. 18790736 10.1074/jbc.M806041200 PMC2576549

[21] Kondo N, Toyama T, Sugiura H, Fujii Y, Yamashita H (2008) miR-206 Expression is down-regulated in estrogen receptor alpha-positive human breast cancer. Cancer research 68: 5004–5008. 18593897 10.1158/0008-5472.CAN-08-0180

[22] Garcia AI, Buisson M, Bertrand P, Rimokh R, Rouleau E, et al (2011) Down-regulation of BRCA1 expression by miR-146a and miR-146b-5p in triple negative sporadic breast cancers. EMBO molecular medicine 3: 279–290. 21472990 10.1002/emmm.201100136 PMC3377076

[23] Volinia S, Galasso M, Sana ME, Wise TF, Palatini J, et al (2012) Breast cancer signatures for invasiveness and prognosis defined by deep sequencing of microRNA. Proc Natl Acad Sci U S A. 109: 3024–9. 22315424 10.1073/pnas.1200010109 PMC3286983

[24] Lu Z, Ye Y, Jiao D, Qiao J, Cui S, et al (2012) miR-155 and miR-31 are differentially expressed in breast cancer patients and are correlated with the estrogen receptor and progesterone receptor status. Oncology letters 4: 1027–1032. 23162645 10.3892/ol.2012.841 PMC3499613

[25] Huang DW, Sherman B, Lempicki RA (2009) Systematic and integrative analysis of large gene lists using DAVID bioinformatics resources. Nature Protocols 4: 44–57. 19131956 10.1038/nprot.2008.211

[26] Rhodes DR, Chinnaiyan AM (2005) Integrative analysis of the cancer transcriptome. Nat Genet 37: S31–37. 10.1038/ng1570 15920528

[27] McLachlan GJ, Bean RW, Peel D (2002) A mixture model-based approach to the clustering of microarray expression data. Bioinformatics 18: 413–422. 11934740 10.1093/bioinformatics/18.3.413

