# Supplementary material for: Unraveling the Hidden Heterogeneities of Breast Cancer Based on Functional miRNA Cluster
Source: PLoS One. 2014 Jan 30;9(1):e87601. doi: 10.1371/journal.pone.0087601 (PMC3907466; doi:10.1371/journal.pone.0087601)
Supplement: Table S1 — The correlation coefficient of among miRNAs in . (DOCX) [file pone.0087601.s001.docx]

**Supplementary I**

Table S1 The correlation coefficient of among miRNAs in $mG_{4}$

|  | miR-103 | miR-107 | miR-142-3p | miR-142-5p | miR-146a | miR-146b | miR-150 | miR-155 | miR-221 | miR-222 | miR-145 | miR-143 | miR-7 |
| --- | --- | --- | --- | --- | --- | --- | --- | --- | --- | --- | --- | --- | --- |
| miR-103 | 1 | .967^**^ | .039 | .105 | .065 | -.025 | .010 | .018 | .374^**^ | .429^**^ | .476^**^ | .048 | .088 |
| miR-107 | .967^**^ | 1 | .020 | .123 | .040 | -.020 | -.022 | .000 | .367^**^ | .437^**^ | .440^**^ | .050 | .100 |
| miR-142-3p | .039 | .020 | 1 | .704^**^ | .781^**^ | .502^**^ | .762^**^ | .610^**^ | .384^**^ | .283^*^ | .264^*^ | .119 | .314^**^ |
| miR-142-5p | .105 | .123 | .704^**^ | 1 | .565^**^ | .475^**^ | .615^**^ | .363^**^ | .298^*^ | .205 | .140 | .147 | .236^*^ |
| miR-146a | .065 | .040 | .781^**^ | .565^**^ | 1 | .646^**^ | .651^**^ | .475^**^ | .390^**^ | .281^*^ | .431^**^ | .131 | .292^*^ |
| mIR-146b | -.025 | -.020 | .502^**^ | .475^**^ | .646^**^ | 1 | .394^**^ | .296^*^ | .517^**^ | .510^**^ | .351^**^ | .435^**^ | .208 |
| miR-150 | .010 | -.022 | .762^**^ | .615^**^ | .651^**^ | .394^**^ | 1 | .377^**^ | .151 | .021 | .109 | .175 | .303^*^ |
| mR-155 | .018 | .000 | .610^**^ | .363^**^ | .475^**^ | .296^*^ | .377^**^ | 1 | .442^**^ | .216 | .218 | .149 | .199 |
| mIR-221 | .374^**^ | .367^**^ | .384^**^ | .298^*^ | .390^**^ | .517^**^ | .151 | .442^**^ | 1 | .686^**^ | .579^**^ | .332^**^ | -.002 |
| miR-222 | .429^**^ | .437^**^ | .283^*^ | .205 | .281^*^ | .510^**^ | .021 | .216 | .686^**^ | 1 | .544^**^ | .328^**^ | .088 |
| miR-145 | .476^**^ | .440^**^ | .264^*^ | .140 | .431^**^ | .351^**^ | .109 | .218 | .579^**^ | .544^**^ | 1 | .366^**^ | .008 |
| mIR-143 | .048 | .050 | .119 | .147 | .131 | .435^**^ | .175 | .149 | .332^**^ | .328^**^ | .366^**^ | 1 | .062 |
| miR-7 | .088 | .100 | .314^**^ | .236^*^ | .292^*^ | .208 | .303^*^ | .199 | -.002 | .088 | .008 | .062 | 1 |

**. Correlation is significant at the 0.01 level (2-tailed).

*. Correlation is significant at the 0.05 level (2-tailed).
