# Supplementary material for: Unraveling the Hidden Heterogeneities of Breast Cancer Based on Functional miRNA Cluster
Source: PLoS One. 2014 Jan 30;9(1):e87601. doi: 10.1371/journal.pone.0087601 (PMC3907466; doi:10.1371/journal.pone.0087601)
Supplement: Table S2 — The anti-association miRNA-mRNA of among miR-146b. (DOCX) [file pone.0087601.s002.docx]

**Supplementary II**

Table S2 The anti-association miRNA-mRNA pairs of miR-146b

| Serial No. | mRNA gene name | CC<-0.4 |
| --- | --- | --- |
| 1 | HIVEP2 | -0.567860372 |
| 2 | KIT* | -0.559353655 |
| 3 | CCNA2 | -0.549798251 |
| 4 | TRAF6* | -0.548652742 |
| 5 | NFE2 | -0.507931913 |
| 6 | MESDC2 | -0.5072279 |
| 7 | CARD10* | -0.50184444 |
| 8 | STARD5 | -0.500220963 |
| 9 | CCBP2 | -0.488769165 |
| 10 | LRRC15 | -0.487826255 |
| 11 | SLC26A7 | -0.486869641 |
| 12 | MEP1A | -0.473907345 |
| 13 | PAPOLG | -0.472576921 |
| 14 | ADARB1 | -0.470604586 |
| 15 | COL13A1 | -0.46558524 |
| 16 | HADHB | -0.464899423 |
| 17 | SUMO3 | -0.461073498 |
| 18 | SLC10A3 | -0.460959689 |
| 19 | USP3 | -0.460616475 |
| 20 | TCEAL2 | -0.4570863 |
| 21 | FLJ14054 | -0.456456071 |
| 22 | COASY | -0.449454455 |
| 23 | EGR3 | -0.4474842 |
| 24 | ARHGAP6 | -0.446620251 |
| 25 | PHYHD1 | -0.445959305 |
| 26 | TAF2 | -0.445144562 |
| 27 | TXNDC9 | -0.444365291 |
| 28 | ANKRD28 | -0.442801828 |
| 29 | CETN3 | -0.442672215 |
| 30 | ORC5L | -0.441572186 |
| 31 | SH3BGR | -0.441135361 |
| 32 | PMCHL2 | -0.440368651 |
| 33 | COL13A1 | -0.436858973 |
| 34 | EPHA5 | -0.434296138 |
| 35 | LOR | -0.433309529 |
| 36 | CCNA2 | -0.432386762 |
| 37 | KIAA0460 | -0.430515285 |
| 38 | BMP7 | -0.430451993 |
| 39 | HNRPD | -0.42935155 |
| 40 | ADCY9 | -0.429320142 |
| 41 | PDCD11 | -0.429064354 |
| 42 | F5 | -0.428724859 |
| 43 | EIF3S1 | -0.428110033 |
| 44 | TGIF | -0.42760719 |
| 45 | ZNF37A | -0.427069017 |
| 46 | CCNB3 | -0.425863624 |
| 47 | RNPEP | -0.425693295 |
| 48 | WRN | -0.425597713 |
| 49 | KIAA0133 | -0.424753946 |
| 50 | TOMM34 | -0.424548689 |
| 51 | BCORL1 | -0.424347681 |
| 52 | NP | -0.423798603 |
| 53 | CCT3 | -0.422840746 |
| 54 | FTHL17 | -0.421934918 |
| 55 | GRIA3 | -0.418463232 |
| 56 | CXXC1 | -0.418250703 |
| 57 | ZNF160 | -0.416394933 |
| 58 | DTNA | -0.415458799 |
| 59 | DHX15 | -0.414969249 |
| 60 | PIP5K2B | -0.413373516 |
| 61 | B3GAT3 | -0.413140561 |
| 62 | CCNA2 | -0.40984761 |
| 63 | CGA | -0.409776591 |
| 64 | KIAA1622 | -0.408811334 |
| 65 | C10orf86 | -0.408690291 |
| 66 | LACTB | -0.407599152 |
| 67 | MLF1 | -0.40749053 |
| 68 | SFRS9 | -0.405972263 |
| 69 | TRGV9 | -0.405410572 |
| 70 | RG9MTD1 | -0.405389395 |
| 71 | COL13A1 | -0.405383448 |
| 72 | SLC38A6 | -0.404380186 |
| 73 | CTPS2 | -0.404261999 |
| 74 | BCCIP | -0.403426034 |
| 75 | CNTFR | -0.402687342 |
| 76 | COPS3 | -0.400606431 |

*. Experimently validated target genes of miR-146b by miRTarBase.
